# Ectopic Cushing’s syndrome in a patient with inferior petrosal sinus sampling indicating pituitary‐dependent ACTH secretion

**DOI:** 10.1002/ccr3.2586

**Published:** 2019-12-14

**Authors:** Alexander Micko, Stefan Wolfsberger, Engelbert Knosp, Wolfgang Marik, Christine Haberler, Yvonne Winhofer, Anton Luger, Greisa Vila

**Affiliations:** ^1^ Department of Neurosurgery Medical University Vienna Vienna Austria; ^2^ Department of Biomedical Imaging and Image‐guided Therapy Division of Neuroradiology and Musculoskeletal Radiology Medical University Vienna Vienna Austria; ^3^ Institute of Neurology Medical University Vienna Vienna Austria; ^4^ Department of Internal Medicine III Division of Endocrinology and Metabolism Medical University Vienna Vienna Austria

**Keywords:** Cushing's syndrome, ectopic, inferior pertrosal sinus sampling, pituitary adenoma

## Abstract

In an unclear case of Cushing's syndrome, IPSS identifies the origin of ACTH secretion, and together with MRI enables the localization of an ectopic corticotroph adenoma in the parasellar or cavernous sinuses region.

## Graphical abstract

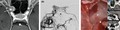

## 1 INTRODUCTION

Endogenous Cushing‘s syndrome is caused by adrenal, pituitary, or ectopic tumors, which lead to cortisol oversecretion, and consequently to several co‐morbidities such as diabetes, hypertension, osteoporosis, myopathy, depression, and infections.^1^ Localization of the tumor‐producing ACTH or cortisol is mandatory, as surgical resection is the treatment of choice.^1^ However, site localization is often challenging, and inferior petrosal sinus sampling might be needed for the differential diagnosis between ectopic and pituitary‐dependent Cushing‘s syndrome.

## 2 CASE REPORT

A 43‐year‐old Caucasian woman was admitted to the endocrinology ward for the diagnostic workup of Cushing's syndrome. She had a 1‐year history of hirsutism and secondary amenorrhea, marked hyperandrogenemia (Table 1) and showed no suppression of cortisol after the administration of 1mg dexamethasone. Co‐morbidities included arterial hypertension and osteoporosis. The past medical history included a sleeve gastrectomy for severe adiposity performed before 11 years ago, and a hip replacement surgery performed five years ago. Her father had type 2 diabetes, the mother had pulmonary hypertension. The patient was a smoker (30 pack‐years), her weight was 60 kg, height was 155 cm, BMI 25 kg/m^2^, and she reported no weight gain during the last 12 months. A proximal myopathy was observed. The 24‐h urinary cortisol excretion was increased (925 µg/24h; normal range 36‐137 µg/24 h) confirming the diagnosis of endogenous Cushing's syndrome. The patient had a hypogonadotropic amenorrhea, but no other pituitary deficiencies (Table 1). Following the administration of corticotropin‐releasing hormone, there was a nearly 2‐fold increase in circulating ACTH, but no significant stimulation of cortisol secretion (Table 2).

**Table 1:** Preoperative laboratory values

Parameter	Value	Normal range
Fasting glucose	93 mg/dL	74‐109
Fasting cholesterol	153 mg/dL	<200
Fasting triglycerides	77 mg/dL	<160
HbA1c	6.1%	4‐6
Cortisol (at 8 am)	16.5 µg/dL	
ACTH (at 8 am)	36 pg/mL	
TSH	0.69 µU/mL	0.44‐3.77
Free T4	1.22 ng/dL	0.76‐1.66
Prolactin	15.3 ng/mL	4.8‐23.3
Luteinising hormone	0.7 mU/mL	7.7‐8.8†
Follicle stimulating hormone	3.6 mU/mL	25.8‐134.8†
17β‐estradiol	15 pg/mL	<49.9
Testosterone	0.77 ng/mL	0.08‐0.48
Bioavailable testosterone	0.28 ng/mL	0.02‐0.22
SHBG	42.1 nmol/L	14.1‐68.9†
Androstenedione	9.57 ng/mL	0.3‐3.7
DHEAS	2.92 µg/mL	0.61‐3.37
17‐OH‐progesterone	1.72 ng/mL	0.3‐2.9

^†^ Normal values in postmenopause.

**Table 2:** Inferior petrosal sinus sampling values

Timepoint (min)	Site	Cortisol (µg/dl)	ACTH (pg/ml)
CRH test			
−15	Peripheral vein	8.6	10
0		7.9	22
15		10.6	26
30		12.9	40
60		11.6	29
90		9.9	5
120		12.2	5
Inferior petrosal sinus sampling: sampling on the way to the inferior petrosal sinus			
	Distally to v. renalis	7.5	27
	At v. renalis	6.3	21
	proximal to v. renalis	7.0	24
	v. cava superior	6.9	50
	v. brachiocephalica dexra	6.9	45
	v. jugulris dextra	8.9	43
	v. brachiocephalica sinistra	7.1	54
	v. jugularis sinistra	9.2	45
Inferior petrosal sinus sampling: CRH test			
−5	Sinus petrosus dextra	6.8	218
0		6.9	194
5		6.8	303
12		6.4	145
20		6.8	81
−5	Sinus petrosus sinistra	7.0	69
0		6.9	25
5		6.6	40
12		6.7	175
20		6.8	94
−5	Peripheral vein	7.2	25
0		7.2	18
5		6.7	24
12		6.4	18
20		6.9	13

We further performed a high‐dose dexamethasone suppression testing administering 4 × 0.5 mg dexamethasone/d at days 1 and 2, followed by 4 × 2 mg dexamethasone/day at days 3 and 4. Although the patient had a previous sleeve gastrectomy, which is shown to accelerate gastric emptying and small intestinal transit, and may impair drug absorption, the test resulted in an about 85% suppression of cortisol to 2.4 µg/dL indicating a corticotroph tumor, but did not significantly impact ACTH (plasma ACTH 15 pg/mL at day 5), diagnosing an ACTH‐dependent Cushing's syndrome.

**Figure 1:**
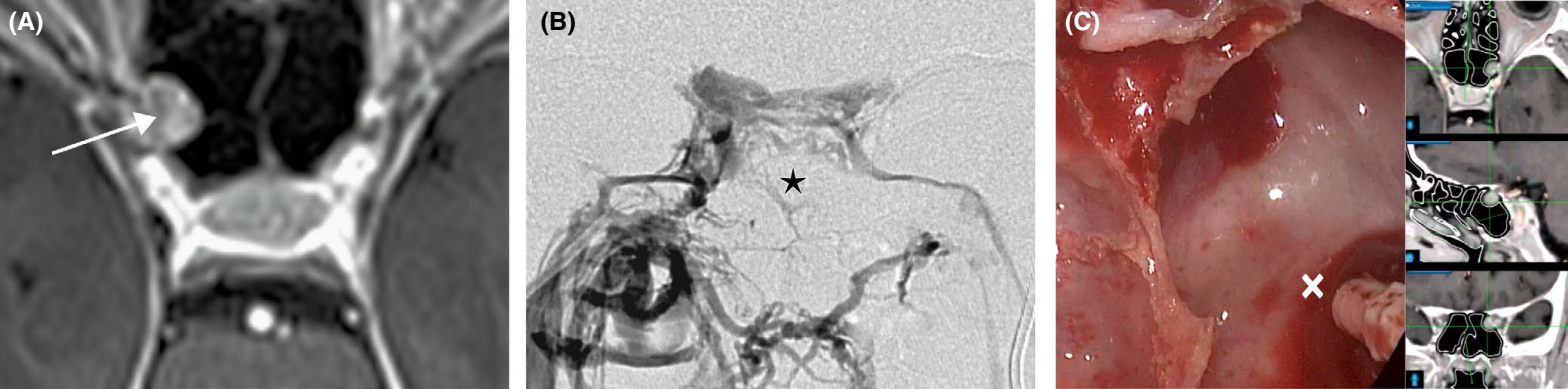
Ectopic corticotroph adenoma in the right sphenoid sinus. A, Axial, contrast‐enhanced MRI of the sphenoid sinus, and sellar region, showing a normal pituitary gland without evidence of any adenoma and a contrast‐enhanced polypoid structure on the right sphenoid sinus wall (arrow). B, Inferior petrosus sinus sampling (contrast agent application through a microcatheter in the right inferior petrosus sinus) showing a vein coming from the polypoid structure in the right sphenoid sinus (asterisk). C, Intraoperative imaging at the time of removal of the polypoid structure in the sphenoid sinus (cross). At the right part of the picture, additional multimodality imaging is provided

We next performed MRI of the sellar region including dynamic images, revealing a normal pituitary gland without any signs of a hypointense mass or a deviation of the pituitary stalk. However, a contrast‐enhancing polypoid structure was found on the right sphenoid wall (Figure 1).

In the context of noticeable ACTH stimulation following the CRH test, but no visible pituitary adenoma in the sella MRI, our pituitary multidisciplinary board decided to perform an inferior pertrosal sinus sampling (IPSS) for the differential diagnosis between pituitary Cushing's disease and ectopic Cushing's syndrome. The IPSS results confirm the central origin of ACTH secretion, demonstrating significantly increased ACTH concentrations in the right inferior petrosal sinus (Table 2).

We next performed an endoscopic transnasal transsphenoidal operation utilizing intraoperative navigation guidance,^2^ inspecting the right endosellar region without finding any signs of a pituitary adenoma and resecting the polypoid mass within the sphenoid sinus (Figure 1).

**Figure 2:**
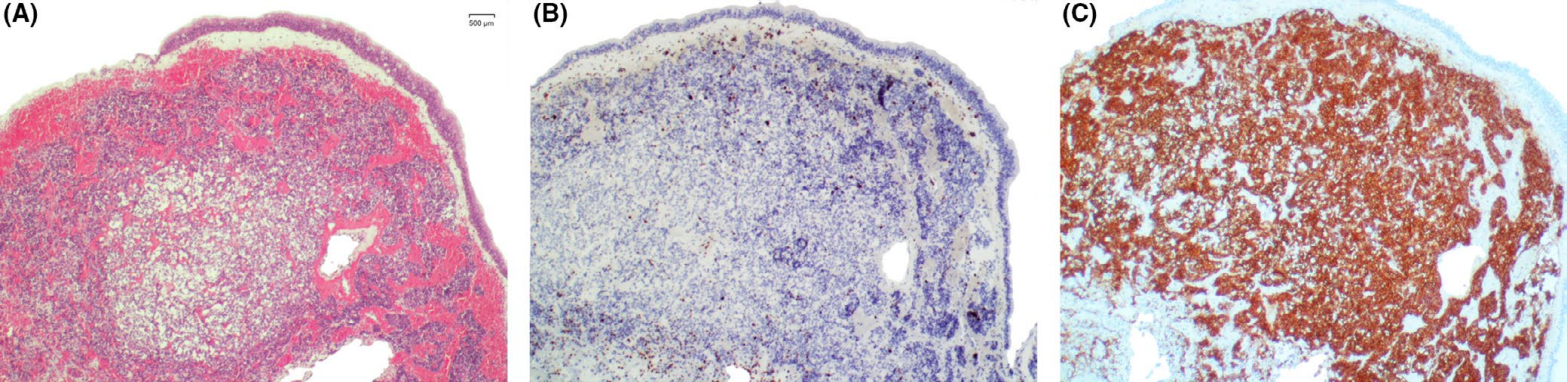
Histopathological examination of sphenoid polypoid structure (40x magnification). A, Hematoxylin and eosin staining. B, MIB‐1 staining. C, Immunohistochemical ACTH staining

Histopathological examinations of the polypoid structure revealed a tumor tissue with a relatively high cell density and a ciliated epithelium on the surface. The entire tissue was covered by a dense blood‐filled capillary network. Mitotic figures were only occasionally seen, MIB‐1 proliferation index was 4.8%. Immunohistochemical examination showed a strong and extensive expression of ACTH, leading to the diagnosis of a corticotroph adenoma located in the right sphenoid sinus (Figure 2).

Three years postoperatively the patient is in remission, last morning cortisol level was 5.7 µg/dL, ACTH 14 pg/mL and 24‐hours urinary cortisol excretion at 4 µg/24 h, with no signs of remaining or recurrent tumor tissue seen on postoperative MRI.

## 3 DISCUSSION

Here, we report a case of Cushing's syndrome due to a histologically confirmed ectopic corticotroph adenoma located in the right sphenoid sinus. To date, only 49 cases of ectopic corticotroph adenomas outside the sella turcica have been reported and are thought to develop from pituitary cells located in the trajectory of Rathke's pouch, the embryogenic structure leading to the anterior pituitary.^3^, ^4^, ^5^, ^6^, ^7^, ^8^, ^9^

Ectopic corticotroph adenomas located in the parasellar or cavernous sinuses behave similarly to anterior pituitary corticotroph adenomas during both stimulatory and suppression tests performed for the differential diagnosis of Cushing's disease.^4^, ^9^ Moreover, inferior petrosal sinuses drain the whole cavernous sinus into the respective internal jugular veins, so IPSS results confirm the central origin of ACTH secretion. This is the reason why ectopic corticotroph adenomas in the parasellar or cavernous sinuses might often be undiagnosed, while remission might be achieved following parasellar irradiation or bilateral adrenalectomy after unsuccessful pituitary surgery.^9^ Corticotroph adenomas often represent microadenomas (<10 mm; 75%‐90%) and cannot be detected in up to 40% by radiological imaging.^3^ Five cases of Nelson's syndrome deriving from a parasellar corticotroph adenoma have been reported following bilateral adrenalectomy.

In the case presented here, the medical history suggests a disease onset 12 months before admission to our department. The patient had arterial hypertension, proximal myopathy, osteoporosis, hirsutism, and secondary amenorrhea. In this case, Cushing's syndrome developed 10 years after sleeve gastrectomy and was associated with neither weight gain nor diabetes, despite previous severe adiposity and a positive family history for diabetes. It appears plausible that the neuroendocrine changes leading to weight loss and diabetes remission after bariatric surgery might also inhibit/prevent the glucocorticoid‐induced changes on energy homeostasis and carbohydrate metabolism; this hypothesis remains to be verified in future studies.

Although a clear MIB‐1 cutoff value has been removed from the current 2017 WHO Classification to identify tumors with an aggressive biological behavior,^10^ a high proliferation index in the presented case indicates a possible tumor recurrence. Although the tumor was resected in toto, the patient will be followed up by yearly clinical examinations and hormone level measurements, as well as with regular MRI controls.

## CONFLICT OF INTEREST

All authors have no financial interest/arrangement or affiliation with one or more organizations that could be perceived as a real or apparent conflict of interest in the context of the article.

## AUTHOR CONTRIBUTIONS

All authors have reviewed and approved the manuscript.
